# Abuse of the Hospital and Its Cure1Read at the First Annual Conference of the British Hospitals Association on September 29, 1910.

**Published:** 1910-12

**Authors:** A. Scott Finnie


### Abuse of the Hospital and its Cure.[1]

By A. SCOTT FINNIE, F.S.A.A.

[*The Hospital*, October 22, 1910.]

SO much has been said and written within recent times with regard to the abuse of hospitals, that one might be excused for thinking that the last word on the subject had been spoken. With those, however, who are convinced that there is abuse, and that it demands rectification, the last word cannot be uttered until effective action has rendered speech unnecessary. It might also fairly be assumed from the attention which the subject has received, that there can be little or no doubt of the actual existence of abuse; and yet, hardly a discussion takes place in which divergence of opinion on this point does not emerge. It may be—indeed, it is very probable—that such diversity of view arises not from dispute as to the actual condition of affairs, but rather from difference in opinion as to what really constitutes abuse.

### WHAT IS A HOSPITAL?

Opinion will depend upon the relative importance in the minds of different people of the varied purposes which the hospital serves. To the average layman the hospital stands first and foremost for charity; while with the hospital medical officer its educational and experimental functions will naturally occupy an important place. The former is mindful to see that the benefits of the charity are confined to suitable recipients, and any departure from this principle is regarded by him as a misuse of his philanthropy. The latter is primarily concerned with the professional needs of the patients. While he is warmly appreciative of the generosity which provides the funds for the hospital, and while he may sometimes entertain a doubt as to whether the treatment of many hospital patients does not constitute a serious encroachment on the livelihood of the general practitioner, the misapplication of funds gifted for benevolence does not so readily occur to him.

Both of these aspects of hospital work—the charitable and the educational—have their own importance; and, in considering the question of the abuse of the hospital, neither must be overlooked, nor must either be allowed to prejudice the other.

While not forgetful of the wider field, and the varying conditions of different localities and individual institutions, it is almost inevitable that a contribution to the discussion of any subject should be largely colored by local circumstances, and to an even greater degree by the personal experience of the contributor. Although what is said will thus specially apply to Scottish hospi-

1. Read at the First Annual Conference of the British Hospitals Association on September 29, 1910.

tals, it will, from the fact that the circumstances of hospitals everywhere are largely similar, and vary only in degree, be capable of general application. It may also be said that, notwithstanding the title of this paper, no pretension is made to offer a complete solution of the problem. The suggestions are offered rather by way of pegs upon which others, riper in experience, may hang their contributions to the discussion.

### THE EXISTENCE OF ABUSE.

The actual position so far as concerns the existence of abuse is so well known to most of those present that any detailed description of it is as unnecessary as it would be uninteresting. It will be sufficient for present purposes to say that the growth of the numbers of those receiving free hospital treatment—as revealed in the statistics published annually—leaves little room for doubt that very many are improperly availing themselves of such treatment, and of the gratuitous services of the medical and surgical staffs.

One or two figures will suffice to indicate the state of affairs in Scotland. In five of the Scottish hospitals the in-patients treated in the year 1889 numbered 21,487. Ten years later the number had increased to 27,320, and last year they totalled 37,056. In twenty years there has thus been an increase of 15,569 in-patients. The out-patients at the same hospitals have increased more rapidly, having advanced from 81,942 in 1889 to 90,791 in 1899, and 150,603 in 1909. In the Aberdeen Royal Infirmary alone the number of in-patients treated in 1901 had increased by 27 per cent. over the figure for 1891, and last year the rate of increase over 1891 was 54.21 per cent. The increases in out-patients for the same periods were 376 and 705 per cent. respectively. The relative increases in the combined populations of the city and county of Aberdeen were approximately 9 and 20 per cent. In view of these figures can it by any possible process of reasoning, or even by any stretch of imagination, be admitted that these increases, at least in the number of out-patients, are due either to inability on the part of the community to pay for medical treatment on the one hand, or to the necessity for obtaining teaching material on the other? It is impossible to attribute these abnormal increases to either of these causes, and, as only on one or other of them can increase disproportionate to the growth of population be justified, the advances are suspiciously suggestive of an abuse of charity.

### CONTRIBUTING CAUSES.

Probably one of the most common reasons why certain people go to a hospital is because others, to all appearance similarly situated, have been there before. They simply follow the crowd, never stopping to inquire as to the propriety of their action. Many also find themselves taking advantage of the hospital through sheer lack of information. One of the commonest misunderstandings is that the members of the hospital staff are remunerated for their services. It is argued that as they are paid from the hospital funds—subscribed in part by the general public—their services should be available when required as a *quid pro quo*. Numbers of people are also found under the impression that the medical and surgical staffs are whole-time servants of the hospitals, and that only there can they be found. It is with the greatest possible surprise that such people hear that Dr. A. or Mr. B. could have advised them equally well in their own consulting-rooms. This conception of the hospital and its honorary staff will doubtless be found most prevalent in remotely situated localities, but the fallacy as to the doctors being paid for their work exists everywhere.

The creation of new departments is inevitably followed by an increase in those attending the hospitals. So long as no specialist is within reach the sufferer will be content with the treatment of the family doctor. The specialist may first become known through his hospital connection. On the formation of the department and his appointment the public become aware that he is to be found at the hospital at certain times. They never inquire whether he can be consulted elsewhere. On his part, the desire to advance the importance of his department may make him less particular as to the suitability of his cases than he might otherwise be and may ultimately become. The department becomes better and better known, and more and more frequented, until it is discovered that many are availing themselves of the specialist's skill under his hospital appointment who are able and ought to be his private patients. It can readily be seen how easily abuse of the hospital is thus created and extended.

Still another cause for abuse is to be found in the aversion of patients to a change of doctor during an illness. Someone comes by an accident and is conveyed to the casualty ward, where first-aid is rendered. The injured thinks it desirable that his treatment should be begun, continued, and ended by the same doctor. Accordingly he attends until recovery, instead of taking his first-aid gratefully and then placing himself under the care of his own medical attendant.

It is sometimes alleged that medical officers of Friendly Societies and Clubs send members to the out-patient departments for treatment on the plea that dressing, when required, can be more satisfactorily performed there. It is, however, hardly conceivable the members of the profession should relieve themselves of patients for whom they are remunerated, to however small an extent, by passing them on for treatment to their professional brethren whose services at the hospital are entirely gratuitous.

Where the hospital forms part of a medical school the professional desire to see as many cases as possible, particularly those of an interesting and informative character, helps to overcome scruples regarding the financial ability of the patients.

The growth in numbers is not infrequently regarded as a sequel to the enlarged financial support now given to the hospitals by bodies of employees, it being alleged that many of the contributors demand treatment for ordinary ailments as a right in return for their subscriptions. This may be true to a very limited extent, but few contributors are ignorant of the fact that practically the whole expenditure of the hospital is upon in-patients, and no question has ever been raised as to the workman's right to admission when he requires it. It may be assumed that the type of man who claims out-patient treatment as a right in return for contributions will be found at the hospital whether contributing or not.

### IN-PATIENT ABUSE.

The causes already mentioned apply more particularly to the out-patient departments, and there can be little doubt that the attendance of many people at those departments, even under misconception, can hardly be regarded as other than abuse. But abuse is alleged against in-patients as well. It is, however, necessary to recognise that an unsuitable applicant in the out-patient department may have a perfectly just right to be admitted as an in-patient. While abuse in the out-patient departments may be asserted with tolerable certainty, its existence amongst in-patients requires to be alleged with caution. The increase in the number of in-patients—though not inconsiderable—may be due, not so much to the invasion of the hospital by the well-to-do, as to a legitimate increase of the class who have always been reckoned worthy of admission. The great advance in medical and surgical science, with the comparative immunity from fatal results after operation, are encouraging people in all ranks to an ever-enlarging degree to submit themselves to treatment. The surgeon prefers,—and that properly—to operate only under the conditions most favorable to the success of his work and the welfare of his patient. Despite improved housing accommodation, and even the presence of a district nurse, he has practically only two alternatives—the public hospital or a private nursing home. With the two extremes of society there is no difficulty. The rich man needs not to hesitate in selecting a home, while the poor man has no choice but the hospital. With these no question of abuse arises, but between them there is the great mass of middle-class people, and among them occurs the greatest so-called abuse of the hospital. so far as it may be found indoors. But how far can it be justly termed abuse? With many, even of them, no doubt can exist as to their suitability for the hospital. Of the remainder, how many can enter a nursing home and pay the usual charges? Comparatively few. They also have no alternative but the public hospital. This is not abuse in the sense of taking improper advantage. It is that they are driven there through force of circumstances, and in the absence of provision appropriate to their measure of ability to pay. If there be a stigma, it rests not on the hospital patient but on the community which fails to make suitable provision for this large and important section of its members in the hour of extremity. Of course, there are people who become in-patients who are able to defray the cost of their treatment elsewhere, but there are black sheep in every flock. No doubt, also, many who are treated in the hospital are singularly unmindful of the benefits received, so far as regards the giving of donations. On the whole, however, it is believed that abuse, so far as concerns in-patients, is due less to intentional sorning upon charity than to sheer lack of any alternative place of treatment.

### THE RESULTS OF ABUSE.

The desirability of reform is emphasised by the resulting effects of the present condition of affairs. First among these may be mentioned the threatened—already in some cases the actual—alienation of the sympathy and financial support hitherto enjoyed by the hospitals. The most liberal givers are usually the most enlightened. With perfect consistency they require to be satisfied as to the genuineness of the objects of their bounty. It therefore becomes increasingly necessary, on financial grounds, that the benefits of the hospital should not be unworthily bestowed.

A second result of present conditions is the deprivation of the medical profession of its legitimate practice and income. It is manifestly unfair that hospital officers, who are also general practitioners, should have to treat gratuitously, in out-patient departments, people perfectly able to pay ordinary medical charges for ailments capable of successful home treatment. It is no less objectionable that the time of specialists should be occupied in attending to the most ordinary and trivial complaints.

Another result is the delay in admission, sometimes the entire exclusion, of really deserving applicants, through the occupation of beds by others less necessitous.

A fourth, and perhaps the most serious result, is the inevitable demoralisation of the community. Dependence upon charity, and the acceptance free of that for which a person should and could pay, are to be discouraged at all costs, not in the interests of charity only but for the sake of the individual. In this respect, the administrators of hospitals do well to see that in extending the aid of charity to heal the body, they are not assisting to propagate a greater evil.

### SUGGESTED REMEDIES.

It only now remains to say something in regard to possible remedies, and the difficulty of offering any suggestions likely to find general acceptance is at once conceded. Indeed the meagre success which has attended previous efforts in this direction invests the attempt almost with a sense of hopelessness. Nevertheless, if the need for reform be admitted, no difficulty, however great, should be allowed to stand in its way. The efforts of those who have in the past attempted to grapple with the problem are by no means forgotten. It may not be amiss to suggest that their failure to accomplish may have been due as much to the lack of united support from their fellow-workers in the hospital world as from the magnitude and complexity of the task.

Abuse arising from ignorance and misconception could possibly be removed or at least minimised by enlightening the public as to the real character and objects of a hospital. As the *personnel* of a community is constantly changing, and memories are short, this would require to be done on some regular system of disseminating information in forms which could not fail to be observed. Much might also be done by judicious officials to prevent the return after a first visit of obviously unsuitable patients.

All this, however, would be but tinkering with the situation. It has to be realised that the present system has become out-worn, not necessarily from any inherent defect in the system itself, but because it has served its day. It is the product of times and conditions totally different from those which now prevail. One of the main obstacles in the way of reform is simple unwillingness to change. It is surprising how many people live as if their eyes were in the back of their heads. They are ever gazing backwards, admiring, and resting content with the achievements of the past, forgetful that these have been but the natural outcome of the circumstances and conditions of the time. The failure to recognise that the conditions of the present are essentially different accounts for the ineffectiveness of much present-day effort. The realisation of this change must precede any real reform. Instead of attempting to patch here and mend there, the whole system has to be remolded in the light of altered times and modern requirements.

### REORGANISATION WANTED.

What is needed is the reorganisation of charitable medical relief on a plan which would, to the fullest possible extent, conserve the interests of the really poor and deserving; under which those partially, but not wholly, able to provide for themselves would be assisted in the manner best calculated to preserve their independence and self-respect; and also under which the members of the medical and surgical staffs could feel that they were neither bestowing their generosity on the undeserving, nor indirectly contributing to the impoverishment of their professional brethren, the general practitioners. Surely it is not beyond the power of the best minds amongst hospital administrators, doctors, and subscribers to construct from the experience of the past and the knowledge of the present a plan of the nature indicated. The success attending the efforts of the administrators of the great London hospital funds in the unification of accounts is an object-lesson in what can be done when men are in real earnest. It may be dangerous to prophesy, but there are not wanting signs that unless reform takes place from within it may come from without, and that not unlikely in a shape which many may regret. Like all other questions, that of nationalising or municipalising the hospital, has more than one side.

REFORM SCHEMES.

The enunciation of some general essentials of reform seems more appropriate to the present occasion than any attempt to arrive at agreement on minute details. Among the first is uniformity of practice in all hospitals engaged in similar work. So long as free-and-easy methods as to admission are allowed to prevail in one institution, perfection in its neighbor is hardly to be looked for. Reform may necessarily include the definition of inability to pay, both as regards outdoor and indoor treatment. This in turn will involve investigation as to the financial position of applicants, but in the hands of wise and tactful officials this need not prove offensive. It would greatly help in this direction if a scale of maximum fees for the treatment of particular ailments could be adopted. By this means the approximate cost of a treatment, either at a patient's own, or a nursing home, could be readily ascertained. The eligibility of applicants would then be determined upon a comparison of financial position with probable cost of private treatment. Has the work become sufficiently standardised to render such a scale practicable? With out-patients it would be a choice of doctors—the hospital medical officer or the general practitioner. With in-patients it would not only be a question of who was to treat, but also where it was to be done. This again raises the perplexing problem of the middle-class in cases where home treatment may be considered impracticable. A solution often suggested is paying wards or beds in the public hospitals. The success of this plan is doubtful. Human nature has to be reckoned with, and unless such patients could be entirely isolated, there might be a reluctance to pay for accommodation similar to that occupied by others free of charge. A more desirable alternative seems to be the establishment of public nursing homes, where patients could be accommodated according to their means, and where they would remain under the care of their own medical men, through whom all fees to specialists would be arranged. There seems no reason why such homes should not form part of the hospital system. Administration expenses would be lessened, and it might be possible to combine with the home a staff of nurses available for private cases and as hospital reserves. It is highly probable that, if such homes had existed in recent years there would have been less ground for the allegation of hospital abuse on the part of this class, and it may be safely predicted that if such accommodation becomes available it will be utilised, even though at considerable cost, by many who would otherwise find their way to the public hospital. Movements towards the provision of such accommodation are being closely followed by those interested.

### SOME IMPORTANT ASPECTS.

Any plan to be successful must be founded on reliable and exact information as to the actual needs requiring to be served. As already indicated, there are the necessitous poor and those who require partial assistance. There is, in addition, the very important question of medical education. Bearing closely on the situation are the operations of Friendly Societies, the Compensation Acts, and the various forms of public medical service, the latest of which—the inspection of school children—may not improbably have a considerable effect on the hospitals. While the Legislature has, in this case, provided for the discovery of disease and defects, it has made no provision for their treatment. A first step in any real reform seems to be conference between representatives of all the interests involved. Subscribers would express their views as to the bestowal of their benevolence. Hospital governors would tell of the difficulties experienced and to be encountered. From workmen's representatives would be obtained valuable aid in determining appropriate standards of inability to pay, keeping in view other provisions for times of sickness. The hospital medical officers, aided by the general practitioners, would guide as to the professional suitability and the best means for regulating the admission of the patients, as well as represent the interests of the profession generally; and the claims of medical education would be looked after by professors. From such conference it should be possible to devise a system under which all legitimate interests would be adequately provided for, and under which abuse would have difficulty in existing. The hospitals would be restored to the position which they should, but at present do not, occupy. From the standpoint of the poor they would be the embodiment of the highest charity; their educational functions would remain unimpaired, and the medical profession would be able to regard them as valued allies in special cases, instead of looking upon them as competitors.

It seems not inappropriate that a lead in the direction indicated should be given by the British Hospitals Association. The hospital administrators of the country are the trustees of a very important section of the national benevolence, and on them rests the responsibility of seeing that it is neither abused nor misapplied.

DISCUSSION.

This paper was discussed in combination with Dr. Loch's paper on "A Social Policy for Hospitals," fully reported in *The Hospital* of October 15.

Col. J. A. Roxburgh (Glasgow): I am quite aware that there must be a great difference of opinion as to whether actual abuse exists, and it may be that abuse, from one point of view, exists, and from another point of view it does not exist; according as we look at the hospital and its intention. It may be that there is abuse from the point of view of the medical practitioner, and no abuse from the point of view of the patient who is admitted. Here in Glasgow, which I happen to know something about, we do not admit that there is much abuse at all. There are certain cases, I daresay, which may be picked out, and which have crept in unawares, but, taken all round, there is not much abuse of hospitals in Glasgow, at any rate from the point of view of the patients. I think we are all agreed that, from the practitioner's point of view, there is, according to the present system, more or less abuse, and I am sure that all of us would welcome paying hospitals where people could go and get the attendance they want under the direction of their own medical man—applause—but when you talk of making a paying department in a general hospital, you get into a great deal of trouble. (Hear, hear.)

PAY WARDS.

Some little time ago, when we were building a new wing in connection with the Western Infirmary, I suggested to one or two of the managers that I thought it would be a good opportunity of trying a paying hospital, and that we might with a moderate charge be able to get from this new wing a sufficient income practically to make it pay itself. Some of the managers thought that it was an excellent idea, but when we began to go into details we found that it was unworkable. First of all the income we have is given to us for the free treatment of needy people, and we could not very well take any part of that to treat those who were agreeable to pay; but further than that, if you are going to have a place of that kind and ask people to pay, each patient would wish his own medical attendant, and then this medical attendance would need to be paid, and then there would be difficulty in the hospital where the medical men in the other part were giving their work gratuitously. It seems to me that the discussion this morning brings out a need of paying hospitals, or something of that kind. We are all very much agreed on that, but for my part, unless we revolutionise the whole system in this part of the country, I do not see how we can make paying wards in our present hospitals. (Applause.)

Mr. W. B. Blaikie (Edinburgh): I would like to say one or two words in corroboration of Col. Roxburgh's remarks. I have listened with extraordinary interest to the remarks from the platform, and I am sure that no one understands the position better than Mr. Thies, and I believe no one has better gripped the exact difficulty of mixing paying and free beds than he has. We have also heard a speech from Professor Jones, who advocates what I might call a great co-operative movement in which medical and surgical assistance will be given to the rich as to the poor, but would not that create one of the difficulties that we are met with and throw out all the junior medical practitioners? Would it not lead to the hospital being a great co-operative store, and would it not be increasing unemployment? It may be that the profession is overstocked, but that is a problem that faces us. It is very pleasant to hear about the German way at Eppendorf, but I am afraid that, knowing human nature as I do and knowing the nature of those to whom we look for subscriptions, the mixture of free and paying beds will fail.

RESOLUTIONS REQUIRED.

Professor Jones: There is a question I would like to put. What about resolutions? It seems to me that there is a considerable body of agreements here, as, for instance, that in some way or other the paying element may be possible. Is it not possible to frame a resolution so as to embody the feeling of this conference?

The Chairman: Well, sir, I would say that I think it is a very dangerous thing to plump a resolution before a meeting which has not had time to consider the matter. (Hear, hear.)

Professor Jones: I quite agree.

The Chairman: If you have any resolution to propose we might consider whether it would be proper to put it to the meeting.

Professor Jones: No, I have not; but what I would suggest is that Dr. Loch and a few others and also the secretary should before the close of this conference consider such a resolution as would gather up the sense of the meeting in order that something practical might follow. I would not propose that right off.

The Chairman: I think that the Council will certainly take your suggestion into consideration, and at all events if they see their way to form a resolution for the consideration of this Association, and give notice of it to-morrow for consideration at the next Conference, that course might be followed. I think that would be the regular way, and I am sure it would be fought out.

### DOES HOSPITAL ABUSE EXIST?

Mr. R. Kershaw (Secretary of the Central London Throat and Ear Hospital): I should like, at the outset of my remarks, to make the deliberate statement that I am not a believer in hospital abuse. I am not a believer that there is a wilful abuse of hospital charity, and that more frequently than not when it does take place it is through ignorance rather than design; but I readily admit that the present state of affairs by which there is an enormous increase in the number of recipients for hospital relief is a condition of affairs worthy of inquiry. When that inquiry has been made, it will, I think, be found that there can be no limitation in the present system of hospital relief, and that the whole system is drifting gradually in the direction of State control, not only of the hospital itself, but in the direction also of a nationalisation of the whole medical service. Medical relief ought not to be a commercial commodity with various standards of excellence. There surely ought to be a uniform system of medical relief for all, and it should be the best that human knowledge can provide whether it is paid for directly by the individual or indirectly through taxation. I know that in making such a statement as this I shall be held guilty of heresy, and I shall be told that the suggestion of State control of hospitals is an ill-return for the endless generosity of our countrymen who by their benevolence have made our hospitals the pride of the country and the envy and admiration of every other. The question of hospital abuse is becoming daily more full of perplexities and consequently more difficut to deal with, and we might well ask ourselves today for a definition of "hospital abuse" so that we may be in agreement that when we use these words we all mean the same thing.

### INDIVIDUALS AND INSTITUTIONS.

There was a time when the acceptance of hospital relief was considered very little removed from the acceptance of parish relief, and the recipients thereof would be made the subjects of commiseration amongst their neighbors, even if a stigma were not placed upon their action. What, then, has brought about such a change of habit when we are now told that the wrong class are securing relief at the hospitals? The answer seems to be obvious that the march of education of the masses on the one side and the enormous progress in medical knowledge and scientific attainment on the other side have had a very natural affinity. These forces in time of need have been brought together, the meeting place has been the hospital, which is the birth-house of medical knowledge and the conveyor of that knowledge to the whole community. The means available in former times for the relief of suffering are now no longer adequate. Knowledge increases very fast, too fast for each individual mind to keep pace with it, and consequently the medical practitioner who enters upon the work of his profession, by which means he has to live, soon finds himself confronted with a most unwilling but powerful competitor.

If we consider the question of hospital abuse from the point of view of the individual, we are at once reminded that we have been taught to believe that we are children of the State which gave us birth. The State has a duty to perform to its citizens, and the foremost duty must surely be in the direction of maintaining healthy citizens. The state demands that we shall be provided with an education, it controls the quality of our food, and it sees that we have pure drugs; it ought, therefore, to provide the means of access to health, while the means must be simple and ought to be adequate. As I have said before, I do not believe that hospital abuse exists in every walk of life, but the sufferer seeks relief at the hospital because he believes that he will derive the greater benefit there, not because he wishes to avoid paying a doctor's fee. Those who are continually crying out about hospital abuse, and denouncing the managers of the hospital for its existence can have little idea of the difficulties which beset them in their endeavor to obviate it.

